# Nutritional Intake Differences in Combinations of Carbohydrate-Rich Foods in Pirapó, Republic of Paraguay

**DOI:** 10.3390/nu15051299

**Published:** 2023-03-06

**Authors:** Yuko Caballero, Konomi Matakawa, Ai Ushiwata, Tomoko Akatsuka, Noriko Sudo

**Affiliations:** 1 Cooperative Faculty of Education, Utsunomiya University, Utsunomiya 321-8505, Japan; 2 School of Pharmacy, Kitasato University, Tokyo 108-8641, Japan; 3 Natural Science Division, Faculty of Core Research, Ochanomizu University, Tokyo 112-8610, Japan

**Keywords:** food frequency questionnaire (FFQ), one-day weighed food record (WFR), dish combinations, carbohydrate-rich dishes

## Abstract

A national strategy for obesity prevention has been promoted in Paraguay, reflecting the situation where half of adults and 23.4% of children (under 5 years old) are overweight. However, the detailed nutritional intake of the population has not yet been studied, especially in rural areas. Therefore, this study aimed to identify obesity-causing factors in Pirapó by analyzing the results from a food frequency questionnaire (FFQ) and one-day weighed food records (WFRs). From June to October 2015, 433 volunteers (200 males and 233 females) completed the FFQ with 36 items and one-day WFRs. Body mass index (BMI) positively correlated with the consumption of sandwiches, hamburgers, and bread and with age and diastolic blood pressure, although pizza and fried bread (pireca) had a negative correlation in males (*p* < 0.05). BMI positively correlated with systolic blood pressure, whereas it negatively correlated with the consumption of cassava and rice in females (*p* < 0.05). The FFQ revealed that fried food with wheat flour was consumed once a day. WFRs showed that 40% of meals consisted of two or more carbohydrate-rich dishes, significantly higher in energy, lipids, and sodium than those containing only one carbohydrate-rich dish. These results imply that less oily wheat dish consumption and healthy combinations of dishes should be considered for obesity prevention.

## 1. Introduction

Obesity is a known risk factor for many diseases, and recent studies have reported that it is caused by globalization and lifestyle urbanization, including eating habits [1,2]. Meanwhile, some studies have reported that a rising rural body mass index (BMI) is a major factor in the global obesity increase in adults [3]. In Latin America, more than half of adults are overweight, significantly higher than the global average of 39% [2]. People in the Republic of Paraguay, located in the center of South America, are affected at the same prevalence and a BMI increase has been noted in both men and women [3,4]. The Instituto Nacional de Alimentación y Nutrición, Ministerio de Salud Pública y Bienestar Social in Paraguay (INAN) reported that 46.4% of pregnant women were overweight, and obesity in females correlated with fewer formal education years [5,6]. Furthermore, a report from the Food and Agriculture Organization (FAO) and other collaborating organizations, including the International Fund for Agricultural Development (IFAD), United Nations International Children’s Emergency Fund (UNICEF), World Food Programme (WFP), and World Health Organization (WHO), noted that the percentage of overweight children (under 5 years of age) in Paraguay was 23.4%, which ranked high among South American countries [7]. Therefore, nutritional education is crucial, though the need for such education is only just beginning to be addressed at the governmental level [8].

The WHO reported that a cause of death from non-communicable diseases in Paraguay accounts for 75% of deaths, higher than the world average of 70%, and that the top three non-communicable diseases that lead to death are ischemic heart disease, stroke, and diabetes mellitus [9]. Regarding non-communicable diseases, 10% of the population has diabetes, and one in three people have hypertension [10,11]. To develop a strategy for preventing obesity and non-communicable diseases at the public level, obesity-related factors, especially eating styles, should be identified and reshaped.

The main staple foods in Paraguay were cassava (mandioca) and various types of corn meal until the 1490s, the years European settlement started, similar to other Central and South American countries. Since then, various other staple foods have been introduced. Regarding wheat flour, an Organization for Economic Cooperation and Development (OECD)-FAO Agricultural Outlook Report, and statistical information derived from the United States Department of Agriculture (USDA), reported that annual domestic wheat consumption in Paraguay has increased drastically, in fact, by nine times over 60 years (from 1960 to 2020), with the consumption per capita approximately 52 kg in 2020 [12,13]; however, cassava and corn are still the primary sources of carbohydrates in rural areas. Meanwhile, fruit and vegetable consumption is low, with 61.9% of males and 71.0% of females not consuming an adequate amount of fruits and vegetables (as the WHO recommends) [14,15].

Nutritional surveillance in Paraguay was first widely conducted in 2011, with limited foods surveyed [11]. At the regional level, there are only a few reports analyzing habitual food consumption and diet patterns in certain areas, especially rural areas [10,16,17,18]. Therefore, national policies for healthy dietary behavior have not yet been established [8,19]. Regarding medical welfare, the national health insurance coverage rate is only 3%, which causes delays in patients’ treatment and worsens their diseases, as was demonstrated during the coronavirus disease 2019 pandemic [4,20]. For geographical and economic reasons, people in rural areas have difficulty accessing medical treatment. Hence, healthy diet education and prevention of obesity and obesity-related diseases in rural areas are crucial. Finding direct causes of obesity and tackling them are ideally required. However, the diet is a complicated part of human lives and requires longitudinal study in a carefully monitored environment.

For these reasons, this study aimed to clarify habitual food consumption, analyze meal styles in detail, and identify obesity-related eating factors in Pirapó, a rural area, to compile nutritional information that will help promote obesity prevention.

## 2. Materials and Methods

### 2.1. One-Day Weighed Food Records

This is the second report of our research conducted, and the selection criteria and study design have been described elsewhere [21]. In short, 200 households in Pirapó completed a one-day weighed food records (WFR) at some point from June to October 2015. To determine the study site, we selected Pirapó as the rural area because, although most of its population comprises farmers, there are supermarkets in the area, spread out at 3–10 km distances, and food access is easy. In addition, it was safe to have people complete WFRs in the region when public security was considered.

We recruited volunteers who would participate in the survey by conducting house-to-house visits in both central and suburban areas of Pirapó, accompanied by acquaintances from city offices. The selection criteria were as follows: at least two adults (18–69 years old, any gender) living in the household, who did not restrict their diet or have any health problems requiring food restriction. The population of Pirapó is approximately 7000. Therefore, we recruited 200 households, which was judged to be a suitable sample size for the food survey.

The study was conducted in accordance with the Declaration of Helsinki and approved by the Ethical Committee of Ochanomizu University (no. 2015-14, June 2015). Informed consent was obtained from all participants involved in the study.

The day before the food survey, researchers or their assistants (local residents) explained the survey and acquired informed consent. Height and weight were measured using a portable stadiometer and a weight scale with light clothes and without socks in their home (stadiometer: seca213, Seca, Chiba, Japan, weight scale: HD-660, Tanita, Tokyo, Japan). The participants’ weight and height were measured to calculate their BMI (kg/m^2^). On the day of the WFRs, the researchers and their assistants visited the household in the early morning, within an hour of the participants’ waking time, and their blood pressure was measured three times while in a sitting position (after micturition and before breakfast). Blood pressure was measured using electronic sphygmomanometers (HEM-7200, Omron, Kyoto, Japan), with it taking one minute for each reading. The average of the second and third measurements was adopted as the pressure value. Hypertension was defined as systolic blood pressure >140 mmHg or diastolic blood pressure >90 mmHg [22]. The WFRs were completed as described in our previous studies [23,24]. All foods and dishes, including beverages consumed at each meal, were weighed using digital cooking scales (TL-280, KD-171, Tanita, Tokyo, Japan), and nutritional intakes were calculated in accordance with the food composition table. The survey was conducted by researchers and their assistants who were trained for three days before the study on how to explain the study protocol and demonstrate the correct use of the scale and calculation of the consumption of each food. Four groups, each consisting of two data collectors, comprising a researcher and their assistant, visited each household and stayed all day for the WFRs.

### 2.2. Food Frequency Questionnaire

Habitual food consumption was assessed using the food frequency questionnaire (FFQ). To develop the questionnaire, a year before the study, we conducted a pilot study of WFRs in nine households, created an FFQ list, and asked Paraguayan nutritionists to add items to cover all foods and dishes consumed in the region. Following a previous study, we developed a non-quantitative FFQ (without portion size (g) of the food or dish) because of the lack of validation and reproducibility of quantitative information [25]. Foods and dishes with 32 items were listed in the FFQ, which was pretested by some villagers, then revised to fit them. We confirmed that there were few seasonal variations in the region based on residents’ interviews and our previous survey. The participants in the FFQ were the same people as those who completed WFRs, who were asked to answer how often they consumed the listed foods or dishes on a daily basis. The questionnaires were paper-based and filled out by trained assistants. The question and answer were as per the following example: “How often do you eat bread?” and “I eat it once a day.” Then, the assistant circled “day” from the options of month/week/day, and wrote the number “1” as the frequency. The FFQ values were calculated as numeric numbers; if a participant’s egg consumption FFQ value was 1, it meant that the participant consumed it once a day.

### 2.3. Dish Combination Analysis

To clarify the types of dishes that were combined in a meal, dish combinations were scrutinized. Recipe data were obtained from the WFRs [21]. Energy and other nutritional intakes per dish were calculated based on the food composition tables provided online by the INAN, FAO, and governmental databases of neighboring countries (Argentine, Chile, and Brazil) [26]. Table S1 shows the dishes that appeared in the WFRs in the study and their nutritional characteristics, as calculated from the food composition tables. Some have already been reported in our previous study in Japanese [21].

**Figure 1:**
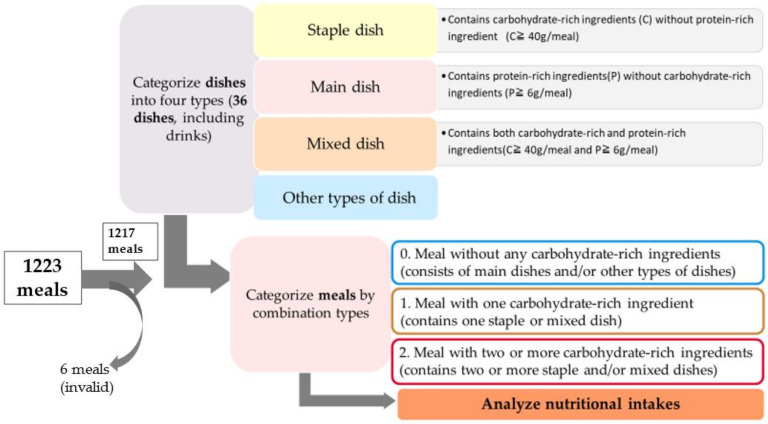
Flowchart of dish combination analysis categorized by meal type.

Figure 1 shows the dish combination analysis procedure. These categorical criteria were based on Murakami and Yoshiike’s study and dietary guidelines for Japanese people because no studies on the combination of dishes were found in other Central and South American countries [27,28].

Among the 200 households, two were excluded because there were no adults aged between 18 and 69 years. So, we collected data on 1223 meals from 198 households. All dishes were categorized into four types: staple, main, mixed, and others. Then, all meals were categorized as follows: one carbohydrate-rich dish, two or more carbohydrate-rich dishes, and not containing any carbohydrate-rich dish.

### 2.4. Statistical Analysis

Multiple regression analysis was used to identify obesity factors and independent variables associated with BMI (the dependent variable) separately for males and females. These factors were selected via the backward stepwise selection method using *p* = 0.05 (both included and removed). In addition to investigating the relationship between blood pressure and the total FFQ values of fried flour dishes (fried tortilla from wheat flour, fried bread (pireca), fried dough with wheat flour (reviro), and fried dumpling with meat and boiled eggs (empanada)), multiple regression analysis was performed. Differences in energy, lipid, and sodium quantities between meal types were analyzed using a *t*-test. The level of significance was set at *p* < 0.05. Statistical analyses were performed using SPSS (Statistical Package for the Social Sciences) version 27 for Windows (IBM, Armonk, NY, USA), JMP version 16.2 (SAS Inc., Cary, NC, USA), and free software R (version 4.2.1; 23 June 2022).

## 3. Results

### 3.1. Participants’ Characteristics

A total of 433 volunteers participated in this survey (200 males and 233 females). Table 1 shows the participants’ physical characteristics, as referred to in our previous study [21]. Approximately 40% of the male participants were farmers, and 70% of the females were housewives. The mean BMI of the participants was 26.4 (standard deviation (SD) = 4.3) for males and 27.4 (SD = 5.2) for females. Overweight and obesity accounted for 61.5% of males and 65.3% of females, respectively.

### 3.2. FFQ

Table 2 shows the FFQ values and the ordered high-frequency intake of food or dishes per day. Any items in the gray color contained wheat flour, accounting for more than half of all food and dishes. The value of boiled cassava was 0.96, both in males and females, reflecting that it was consumed most frequently, approximately once a day. The second and fourth most abundant foods were bread and reviro, respectively; both of which contained wheat flour.

Galleta is a type of small and ball-shaped bread containing anise seed (herb), and reviro is a typical regional meal cooked like a scrambled egg with wheat flour, water, oil, and salt, but without eggs. Tortillas and pireca are made from wheat flour and fried in large amounts of oil. Pireca is fried dough like thin-crust pizza made from wheat flour, salt, and water, sometimes with egg, without fermentation.

The FFQ results and nutrient compositions of the dishes showed that the participants consumed cassava-based and oil-rich dishes with wheat flour (reviro, tortilla, pireca, or empanada) once a day.

### 3.3. BMI and Obesity-Related Factors

Table 3 shows the results of the multiple regression analysis to clarify the relationship between BMI and the independent variables selected via the stepwise method for males. The results showed that age, sandwiches, hamburgers, diastolic blood pressure, and bread (pan, galleta) were significantly positively correlated with BMI, with *p* < 0.001, 0.015, 0.006, 0.017, and 0.018, respectively. In contrast, pizza and fried bread (pireca) were negatively correlated with BMI (*p* = 0.015 and 0.036, respectively).

Table 4 shows the results for females when using the same multi-regression analysis method. Systolic blood pressure was significantly positively correlated with BMI, with *p* < 0.001. However, the consumption of dishes with cassava, or rice was significantly negatively correlated with BMI, with *p* < 0.001 and 0.002, respectively.

Table 5 shows the results of the multi-regression analysis to clarify the relationship between systolic blood pressure and the total FFQ value of fried wheat flour dishes for males. Diastolic blood pressure, age, and total FFQ value of fried flour dishes were significantly positively correlated with systolic blood pressure, with *p* < 0.001, < 0.001, and 0.004, respectively.

For females, no association was found between blood pressure and the total FFQ value of fried flour dishes.

### 3.4. Food Combination Analysis

Table 6 shows the dish combinations observed frequently and their mean energy intake. The average energy intake during lunch was 980 or 780 kcal for males and females, respectively, making the highest contribution to the daily energy intake. However, combinations of reviro with cocido for breakfast and boiled cassava with tortilla for dinner also formed a high energy intake for both males and females. Cocido is a traditional Paraguayan beverage cooked from mate leaves caramelized with sugar and added to water and milk for serving, which is frequently consumed for both breakfast and dinner.

Most of the participants used soybean oil for cooking. Skipped meals were observed for all meals. As the main culprit, approximately 10% of the participants skipped breakfast, 25 males and 24 females.

Table 7 shows the types of dish combinations and the differences in energy, lipid, and sodium intake between the dish types. Only 10 males and 14 females consumed meals without carbohydrate-rich dishes, and their energy and other nutritional intakes were lower than those of individuals who consumed other meal types. Among meal types, 43.8% of males and 40.9% of females consumed meals with two or more carbohydrate-rich dishes. When comparing meals with one carbohydrate-rich dish and those with two or more carbohydrate-rich dishes, energy, lipid, and sodium intakes were significantly higher for meals with two or more carbohydrate-rich dishes (*p* = 0.001 or < 0.001), except for lipid intake in males (*p* = 0.197).

**Figure 2:**
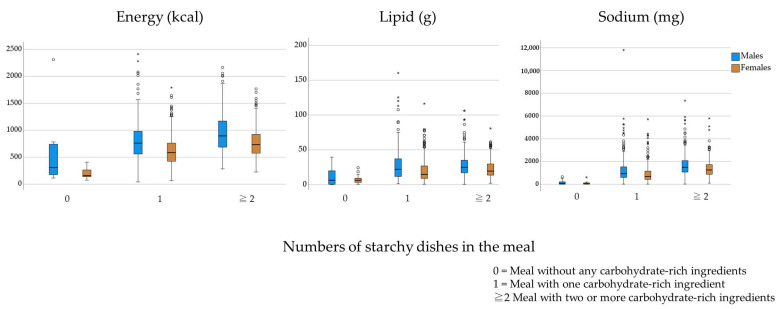
Differences between meal types in energy, sodium, and lipid quantities. Interquartile range (IQR) means from 25th to 75th percentile drawn as the length of the boxes. The upper whiskers represent adjacent values that are not more than 75th percentile + 1.5 × IQR, and the lower whiskers represent adjacent values that are not less than 25th percentile – 1.5 IQR. Stars represent extreme values (larger than 75th percentile + 3.0 × IQR or smaller than 25th percentile – 3.0 × IQR). White circles represent outliers: outside of whiskers, but not extreme values.

Figure 2 shows the distribution of nutritional intake between the meal types. Males showed large ranges in all forms of nutritional intake, especially energy and lipids, for meals with one carbohydrate-rich ingredient, and sodium in meals with two or more carbohydrate-rich ingredients. An outlier in a meal without carbohydrate-rich ingredients in males came from heavy alcohol consumption.

## 4. Discussion

In this study, we scrutinized FFQ values and WFRs to identify obesity-related factors among villagers in Pirapó, Paraguay.

Most participants in Pirapó cultivated cassava in their fields. Therefore, boiled cassava was consumed more frequently than any other food on a daily basis (approximately once per day).

Bread and other staple dishes containing wheat flour were also frequently consumed. Moreover, six of the top ten most frequently consumed dishes contained wheat flour, and they were consumed as staple and main dishes. Among wheat flour dishes, reviro, tortilla, pireca, and empanada contained more than 20 g of lipid, and the sum of these FFQ values reached approximately 1.0 for both males and females. This indicates that they consumed oil-rich flour dishes once daily.

When obesity factors were compared between males and females, the FFQ values for sandwiches, hamburgers, and bread (pan and galleta) were positively correlated with BMI in males. In contrast, dishes with cassava and rice were negatively correlated with BMI in females. The total FFQ value of fried wheat flour dishes was positively associated with systolic blood pressure in males. However, this was not observed in females. Some studies have reported that males tend to be less aware of their body weight and less likely to go on a diet than females [29,30]. Our results could not reveal sex differences at the conscious level. However, considering a previous study conducted in Paraguay, there might have also been a different perspective on food between the sexes in this study [6]. These results imply that different approaches to nutritional education between sexes are needed.

In their review, Gadiraju et al. reported that more frequent consumption of fried foods (i.e., four or more times per week) was associated with a higher risk of developing type 2 diabetes, obesity, and hypertension [31]. Hypertension and obesity are strongly correlated [22,32]. Another cohort study showed that participants who consumed fried foods four or more times per week were approximately 1.2 times more likely to have hypertension than those who consumed fried foods less than twice per week (hazard ratio 1.21, 95% confidence interval 1.04, 1.41) with a median follow-up of 6.3 years [33]. Furthermore, Soriguer et al. reported that a high-frequency intake of fried food, especially reused sunflower oil, contributed to hypertension [34]. At our study site, 38.0% of males and 29.6% of females had high blood pressure, and most participants used soybean oil. The fatty acid composition of soybean oil is similar to that of sunflower oil (native species), which contains a high level of linolenic acid. Therefore, considering the previous report and our results showing a positive correlation between high-frequency intake of fried food and systolic blood pressure [33,34], high-frequency consumption of fried food in this area might contribute to the participants’ obesity and hypertension.

Susceptibility to non-communicable diseases (NCDs), such as hypertension and diabetes, is known to depend on ethnicity and genotype. We did not study participants’ ethnicities. However, considering that approximately 85% of Paraguayans are mestizo and the relationship between genotypes and NCDs has not been studied widely in South America, further study on the relationship between them is required. The average FFQ values of vegetables ranged from 0.44 to 0.45 and their consumption corresponded to 3.08–3.15 times per week, which was higher than the average consumption of vegetables nationally of 2.6 [11]. Nevertheless, vegetable consumption was still low compared with the WHO’s recommendation of five servings of fruits and vegetables on a daily basis, although only a few countries achieve that recommendation [15]. High-frequency consumption of vegetables and obesity are known to be inversely correlated [35]. Our finding of a few varieties of vegetable dishes from WFRs and low-frequency consumption of vegetables from FFQ implies that promoting cooking a variety of vegetable dishes will increase the high-frequency consumption of vegetables and will be effective for preventing obesity in the future. Considering food access, when supermarkets are situated at a distance of 3–10 km, growing vegetables at home could also be an effective measure to promote a greater quantity and frequency of vegetables’ consumption.

According to the WFRs results, meals with bread or dishes with wheat flour and mate milk tea were frequently observed to be consumed during breakfast and dinner. Boiled cassava and meat soup or pasta were observed more frequently during lunch. From a nutritional perspective, even though the participants’ energy intake was sufficient for breakfast and dinner, other nutrients, such as vitamins and minerals, were lacking, with lunch the main source of protein, vitamins, and minerals. Furthermore, a highly refined and small-particle starchy diet is known to induce both de novo lipogenesis and stearoyl-CoA desaturase [36,37]. Therefore, these eating styles of highly refined wheat flour dishes and sweetened mate tea with milk during breakfast and dinner might pose a risk of enlarging adipose cells.

Among the participants, 11% and 6% skipped breakfast and dinner, respectively. However, some participants exceeded the ideal energy intake per meal, particularly when combined with fried dishes, reviro, or tortilla. This might be compensatory behavior for skipping meals and is known to be an obesity factor [38]. Hence, eating meals without skipping could be crucial to covering nutritional necessities and preventing obesity, as the INAN recommends [39].

When categorizing meal types into the number of carbohydrate-rich dishes, meals consisting of two or more carbohydrate-rich dishes accounted for approximately 40% of all meals. When comparing meal types, the energy, lipid, and sodium intakes were significantly higher when consuming two or more carbohydrate-rich meals. While many studies analyzing dietary patterns have been reported worldwide, there are few reports on carbohydrate-rich dish combinations in meals, except in Japan [40,41,42,43]. Some studies reported that they did not observe any cases of combining two or more staple dishes in a meal [27,44]. This might be because Japanese people are educated in school to eat by combining one type of staple food, a main dish, and some side dishes; school lunches are also served in the same manner. In contrast, meals with bread and pasta or meals with two types of carbohydrate-rich dishes are often seen in many countries, although the degree of the combination has not yet been studied. It seems such a combination of dishes depends on each country’s cultural background and nutritional education. A study of Hispanic older adults in the United States showed that BMI and waist circumference were related to diet type [42]. Diet patterns observed in our study implied that there were diet-type differences among participants, which might be related to BMI and other health outcomes, while the average daily energy intake and other macronutritional intakes of participants were normal [21]. Carbohydrates are important energy sources for daily activities. However, a combination of other macronutrients, total energy intake, and cooking style may be important for preventing over-intake of food. Further studies analyzing carbohydrate-rich dishes in the diet and the effects of obesity should be conducted.

Although dietary guides recommend eating well-balanced meals [38,45], a recent study showed that the scores for healthy diet styles in Paraguayans were the worst among 11 Latin American countries, and the prevalent diet styles seemed to be far from the heathy diet recommendation, as shown in a study conducted in Asunción, the capital of Paraguay [39,46]. This situation is presumed because the dietary guides advise on the frequency and quantity of food that people are recommended to consume, but not at the dish level [47].

This study had some limitations. First, we conducted only one-day WFRs, meaning day-to-day variations were not considered. Second, the FFQ was self-reported, and the accuracy depended on participants’ memories. Furthermore, the FFQ did not include portion sizes, meaning we could not calculate the habitual nutritional intake and could not conduct a direct comparison with the WFR. Third, we were unable to conduct a physical energy expenditure survey, though that is an obesity factor. Studies combining food intake and physical energy expenditure must be conducted in the future.

Nevertheless, this is the first report to reveal habitual food intake and dish combinations in detail and identify new routes for preventing obesity. The study’s findings lead us to make to some suggestions. First, people should consume fewer fried dishes, such as reviro, tortilla, and empanada, eating those only a few times per week at most. Second, the consumption of one carbohydrate-rich dish per meal is recommended. If two or more carbohydrate-rich dishes are combined in a meal, care must be taken to avoid over-intake of energy, lipids, and sodium. For example, the ideal eating style for one meal is as follows:

A. Combination of staple dish, main dish, and one portion of vegetables
  i.e., boiled cassava, meat soup without pasta, and salad
B. One mixed food and one portion of vegetables
  Guiso (a dish containing rice, meat, and vegetables) and salad

However, these recommendations are limited to this area. A further consideration is that the situation may have changed after the study. Tracking the area and wider region, including city districts, may be helpful to propose effective dietary advice.

## 5. Conclusions

Oily and carbohydrate-rich dishes, particularly with wheat flour, were often consumed approximately once a day, and meals combining two or more carbohydrate-rich dishes accounted for approximately 40% of all meals in Pirapó. Therefore, nutritional education on using less oil and healthier dish combinations should be promoted for obesity and non-communicable-disease prevention.

## Figures and Tables

**Table 1:** Physical characteristics of participants.

	Male (n = 200)	Female (n = 233)
Age *^1^ (year)	42.4 ± 14.4 *^2^	38.7 ± 13.7
Height *^1^ (cm)	169.9 ± 6.9	158.5 ± 5.8
Weight *^1^ (kg)	76.1 ± 12.9	68.7 ± 13.2
BMI *^1^ (kg/m^2^)	26.4 ± 4.3	27.4 ± 5.2
BMI < 25	77 (38.5%) *^3^	81 (34.8%)
25 ≤ BMI < 30	80 (40.0%)	81 (34.8%)
BMI ≥ 30	43 (21.5%)	71 (30.5%)
Systolic blood pressure (mmHg)	133.1 ± 20.6	127.0 ± 21.3
Diastolic blood pressure (mmHg)	83.2 ± 14.0	80.1 ± 14.2
Hypertension	76 (38.0%)	69 (29.6%)

*^1^ Referring to the previous study [21]. *^2^ Mean ± SD. *^3^ N (%).

**Table 2:** FFQ values of food and dishes.

	Food or Dish	Males (n = 200)		Females (n = 233)	
		FFQ *^1^	SD	FFQ	SD
1	Boiled cassava	0.96	0.55	0.96	0.58
2	Bread (pan, galleta)	0.63	0.51	0.68	0.54
3	Salad, dish with vegetable	0.45	0.37	0.44	0.35
4	Fried dough with wheat flour (reviro)	0.46	0.36	0.42	0.34
5	Rice, dish with rice (guiso)	0.42	0.25	0.40	0.23
6	Hard bread (coquito)	0.36	0.31	0.42	0.42
7	Meat soup with pasta (caldo de carne)	0.35	0.24	0.33	0.24
8	Meat spaghetti (tallarin, fideo con carne)	0.26	0.22	0.26	0.22
9	Fried tortilla from wheat flour	0.23	0.20	0.24	0.20
10	Meat dish (asado *^2^, milanesa *^3^, marinera *^4^)	0.22	0.17	0.22	0.18
11	Fried bread (pireca)	0.16	0.15	0.17	0.15
12	Bean soup (caldo de poroto, legumbre)	0.17	0.17	0.16	0.16
13	Fried dumpling with meat and boiled egg (empanada)	0.15	0.12	0.15	0.12
14	Sweet bread	0.14	0.14	0.15	0.15
15	Dish with cassava	0.14	0.16	0.14	0.15
16	Cornmeal dumpling soup (bori)	0.13	0.11	0.13	0.11
17	Corn bread with cheese and egg (sopa paraguaya)	0.13	0.10	0.12	0.09
18	Cheese bread with cassava starch (chipa, chipa soó)	0.11	0.14	0.11	0.13
19	Dish with corn grain (locro, choclo)	0.10	0.11	0. 09	0.09
20	Cheese pancake with cassava starch (mbeyu)	0.09	0.11	0.11	0.22
21	Pizza	0.06	0.12	0.06	0.10
22	Meatball soup (albondiga)	0.06	0.07	0.05	0.06
23	Sandwich	0.05	0.13	0.06	0.13
24	Doughnut (bollo, rosquilla)	0.04	0.07	0.06	0.13
25	Hot dog (pancho)	0.03	0.06	0.04	0.07
26	Hamburger	0.03	0.06	0.03	0.07
27	Cake (torta)	0.02	0.07	0.03	0.07
28	Cornmeal dish (polenta)	0.02	0.06	0.03	0.07
29	Pudding with wheat flour or bread (budín)	0.02	0.05	0.02	0.05
30	Ñoqui	0.02	0.04	0.02	0.05
31	Sweet dish with cornmeal (polenta dulce)	0.01	0.04	0.01	0.04
32	Lazaña	0.01	0.02	0.01	0.03

Numbers highlighted in gray are dishes with wheat flour. *^1^ FFQ value 1 corresponds to the frequency at which a food or dish is consumed once a day. For example, if the FFQ value is 0.5, they consume the food or dish every two days. If its value is 0.1, it is consumed every 10 days. *^2^ Asado: barbecued or oven-roasted meat. *^3^ Milanesa: beef cutlet. *^4^ Marinera: deep-fried battered beef.

**Table 3:** Variables selected via best subset selection in multiple regression analyses for males.

Independent Variables	Unstandardized *β*	Standard Error	Standardized *β*	*p*
Intercept	18.215	1.932		<0.001
Age	0.079	0.021	0.267	<0.001
Sandwich	9.286	3.793	0.234	0.015
Hamburger	20.263	7.318	0.218	0.006
Diastolic blood pressure	0.052	0.022	0.168	0.017
Bread (pan, galleta)	1.387	0.579	0.167	0.018
Pizza	−12.395	5.030	−0.268	0.015
Fried bread (pireca)	−4.050	1.913	−0.141	0.036

The dependent variable is BMI. Analysis of 190 male participants, excluding those with missing values.

**Table 4:** Variables selected via best subset selection in multiple regression analyses for females.

Independent Variables	Unstandardized *β*	Standard Error	Standardized *β*	*p*
Intercept	19.594	1.998		<0.001
Systolic blood pressure	0.083	0.015	0.337	<0.001
Dish with cassava	−7.307	2.169	−0.209	<0.001
Rice, dish with rice (guiso)	−4.522	1.435	−0.197	0.002

The dependent variable is BMI. Analysis of 219 female participants, excluding those with missing values.

**Table 5:** Results of multiple regression analyses with the total FFQ value of fried flour dishes for males.

Independent Variables	Unstandardized *β*	Standard Error	Standardized *β*	*p*
Intercept	40.148	7.255		<0.001
Diastolic blood pressure	0.884	0.080	0.602	<0.001
Age	0.317	0.077	0.223	<0.001
Total FFQ value of fried flour dishes	5.927	2.024	0.154	0.004

The dependent variable was systolic blood pressure.

**Table 6:** Combinations of dishes observed frequently in WFRs.

Dish Combinations (n)	Mean Energy Intake (kcal)	
	Males (n = 200)	Females (n = 233)
Breakfast (347)	686 ± 394	563 ± 326
Reviro + mate tea with milk (cocido) (78)	909 ± 248 (35)	758 ± 263 (43)
Bread + mate tea with milk (cocido) (73)	648 ± 267 (30)	537 ± 215 (43)
Hard bread (coquito) + mate tea with milk (cocido) (60)	567 ± 193 (25)	569 ± 241 (35)
Skip (49)	0 (25)	0 (24)
Lunch (438)	980 ± 379	780 ± 286
Boiled cassava + dish with rice, meat, and vegetable (guiso) (66)	895 ± 250 (29)	684 ± 211 (37)
Boiled cassava + meat soup with pasta (50)	789 ± 254 (21)	708 ± 236 (29)
Boiled cassava + meat dish (asado, milanesa, marinera) (23)	737 ± 269 (13)	677 ± 365 (10)
Skip (2)	0 (2)	0 (0)
Dinner (432)	757 ± 416	563 ± 323
Bread + mate tea with milk (cocido) (41)	615 ± 215 (16)	519 ± 245 (25)
Hard bread (coquito) + mate tea with milk (cocido) (28)	645 ± 228 (7)	615 ± 263 (21)
Boiled cassava + tortilla (wheat flour) (24)	1174 ± 378 (10)	803 ± 286 (14)
Skip (28)	0 (11)	0 (17)
Total	2423 ± 704	1909 ± 613

There were 227 patterns from 1217 meals.

**Table 7:** Dish combination types and their mean energy, lipid, and sodium intake differences between meal types.

	Dish Combination Types			
	No Carbohydrate-Rich Dishes	Contained One Carbohydrate-Rich Dish	Contained More Than Two Carbohydrate-Rich Dishes	*p* Value *
Male				
Number of meals (%)	10 (1.8)	306 (54.4)	246 (43.8)	
Energy	557 ± 663	792 ± 345	961 ± 360	<0.001
Lipid	10.5 ± 12.8	26.9 ± 21.3	29.0 ± 17.6	0.197
Sodium	170 ± 237	1180 ± 897	1786 ± 1246	<0.001
Female				
Number of meals (%)	14 (2.1)	373 (56.9)	268 (40.9)	
Energy	204 ± 111	619 ± 281	778 ± 276	<0.001
Lipid	7.7 ± 7.0	19.1 ± 15.4	22.9 ± 13.2	0.001
Sodium	108 ± 158	878 ± 728	1408 ± 796	<0.001

* *t*-test between meals with one carbohydrate-rich dish and those with two or more carbohydrate-rich dishes.

## Data Availability

Data is unavailable due to privacy restrictions.

## Supplementary Materials

The following supporting information can be downloaded at: https://www.mdpi.com/article/10.3390/nu15051299/s1; Table S1. Dishes noted through the one-day weighed food records (WFRs) survey in Pirapó and their nutritional compositions.
