# Supplementary material for: Nutritional Intake Differences in Combinations of Carbohydrate-Rich Foods in Pirapó, Republic of Paraguay
Source: Nutrients. 2023 Mar 6;15(5):1299. doi: 10.3390/nu15051299 (PMC10004760; doi:10.3390/nu15051299)
Supplement: Supplementary file 1 [file nutrients-15-01299-s001.zip › nutrients-2231391-supplementary.pdf]

Table S1. Dishes observed at the one-day weighed food records (WFRs) survey in Pirapó and their nutrient compositions[*1].

Food or dish	Portion size (g)	Energy (kcal)	Protein (g)	Lipid (g)	Sodium (mg)
Boiled cassava	178.5 ± 88.8	309.5	1.9	0.3	24.7
	143.8 ± 72.7	192.9	1.5	0.3	19.5
Bread (pan, galleta)	126.4 ± 66.9	363.2	10.4	4.2	609
	108.0 ± 61.5	311.2	8.8	3.6	538
Fried dough with wheat flour (reviro)	201.7 ± 66.1	696.5	15.1	24.5	1048
	162.6 ± 62.6	582.4	12.1	21.3	808
Rice, dish with rice (guiso)	415.9 ± 165.2	594.8	34.1	21.2	1352
	332.5 ± 136.7	477.8	27.0	16.8	1158
Hard bread (coquito)	86.1 ± 45.1	322.1	7.7	3.6	402
	92.0 ± 56.1	347.1	8.5	3.9	424
Meat soup with pasta (caldo de carne)	439.9 ± 148.1	569.3	37.8	22.4	1470
	391.3 ± 143.4	521.7	35.1	19.4	1390
Meat spaghetti (Tallarin, fideo con carne)	487.8 ± 47.4	751.8	51.9	25.9	1164
	354.3 ± 69.3	576.5	40.2	19.8	861
Tortilla from wheat flour	267.4 ± 83.2	793.7	18.6	29.7	1343
	168.2 ± 55.2	546.2	13.2	19.1	975
Cutlet or deep-fried battered beef (milanesa, marinera)	266.5 ± 193.6	807.1	48.4	36.7	2028
	187.4 ± 80.4	635.9	32.9	30.5	1088
Barbecued or oven-roasted meat (asado)	274.8 ± 100.9	605.5	52.7	40.9	1402
	259.3 ± 128.3	525.5	48.2	34.0	710
Fried bread (pireca)	181.9 ± 96.8	753.6	14.6	29.9	929
	143.4 ± 73.9	568.9	10.2	25.6	660
Bean soup (caldo de poroto, legumbre)	180.2 ± 26.3	482.5	20.0	8.6	935
	329.3 ± 122.8	372.1	14.8	8.5	822
Fried dumpling with meat and boiled egg (empanada)	185.5 ± 85.4	656.1	26.7	39.0	1060
	185.6 ± 105.2	623.6	25.9	35.8	1046
Sweet bread	249.6 ± 59.0	854.8	18.0	33.8	315
	178.0 ± 88.7	670.3	13.6	31.8	343
Cornmeal dumpling soup (bori)	505.1 ± 151.5	759.2	51.1	27.4	1125
	325.1 ± 53.4	431.7	27.6	15.7	700

Table S1. (Continued)

Food or dish	Portion size (g)	Energy (kcal)	Protein (g)	Lipid (g)	Sodium (mg)
Corn bread with cheese and egg (sopa paraguaya)	176.5 ± 40.2	477.9	15.1	25.4	802
	126.0 ± 49.6	321.9	10.2	16.8	586
Cheese bread with cassava starch (chipa, chipa soó)	164.8 ± 79.1	606.6	10.9	23.4	700
	133.5 ± 60.0	507.4	8.9	20.3	568
Locro	417.5 ± 60.1	546.5	33.1	22.7	957
	407.2 ± 100.4	528.8	31.5	21.7	928
Cheese pancake with cassava starch (mbeyu)	216.0 ± 70.8	937.8	15.1	39.1	1139
	157.3 ± 50.0	661.6	10.2	26.8	781
Pizza	160.0 ± 8.5	373.1	13.4	12.1	847
	255.0 ± 7.1	562.9	19.2	18.7	1354
Meatball soup (albondiga)	462.0*2	611.2	48.8	23.4	1538
	356.0 ± 2.8	470.7	23.2	18.0	1083
Sandwich	779.0*2	867.7	27.6	38.4	1997
	387.0*2	856.6	27.2	37.9	1971
Doughnut (bollo, rosquilla)	50.0*2	200.0	3.1	11.2	222
	75.3 ± 42.7	301.3	4.6	16.9	334
Hamburger	402.0 ± 246.1	1094.2	49.6	63.0	1710
	258.0 ± 34.6	702.3	31.9	40.5	1097
Cake (torta)	134.0 ± 100.2	398.0	4.7	19.1	437
	102.5 ± 76.8	427.2	6.9	17.4	159
Mate tea with milk, coffee with milk, and other beverages with	270.7 ± 67.5	215.9	6.9	6.0	91
milk.	243.7 ± 58.3	184.9	6.4	5.5	83
Dish with fish	334.0 ± 196.4	417.4	36.9	26.7	1977
	227.7 ± 59.7	309.3	32.5	17.7	427

*1 Energy and other nutrient compositions of some dishes have already been published in our previous study [21]. Reproduced with permission from Journal of the Japan Dietetic Association; published by Japan Dietetic Association, 2017.

*2 n = 1

Mean ± SD

The upper and lower lines show the average portion size and nutrient intake of males and females, respectively.
